# Tuberculose pulmonaire à Brazzaville en hospitalisation pneumologique: impact du diagnostic tardif à l’infection au VIH sur les anomalies radiographiques

**DOI:** 10.11604/pamj.2016.24.259.9456

**Published:** 2016-07-20

**Authors:** Esthel Lee Presley Bemba, Régis Gothard Bopaka, Régis Moyikoua, Rolland Ossibi-Ibara, Norela Bibiane Ebenga-Somboko, Syn Nerval Toungou, Paunel God’hervé Matondot, Boris Kevin Ossale-Abacka, Franck Hardain Okemba-Okombi, Joseph Mboussa

**Affiliations:** 1 Faculté des Sciences de la Santé, Université Marien NGOUABI, Congo; 2 Service de Pneumologie, CHU de Brazzaville-Congo; 3 Service de Radiologie, CHU de Brazzaville, Congo; 4 Service des Maladies infectieuses et tropicales, CHU de Brazzaville, Congo; 5 Programme National de Lutte contre la Tuberculose (PNLT), Congo

**Keywords:** Tuberculose, VIH, Radiographie, Congo, Tuberculosis, HIV, radiography, Congo

## Abstract

L’objectif de notre étude était de décrire les différents aspects radiographiques de la tuberculose pulmonaire selon le degré de l’immunodépression chez les patients VIH. Nous rapportons une étude rétrospective de 80 patients VIH positif non traités présentant une tuberculose pulmonaire, hospitalisés dans le service de Pneumo-phtisiologie de Brazzaville de Janvier 2013 à Janvier 2014. Notre échantillon était composé de 44 femmes (55%) et 36 hommes (45%) soit un sex ratio de 0,81. La moyenne d’âge était de 37,5±9,17ans, la moyenne du Taux de CD4 était de 153,13±86,6cell/mm^3^. La microscopie des expectorations à la recherche des bacilles acido-alcoolo-résistants était positive dans 75% des cas chez les patients ayant un taux de lymphocytes T CD4>200cell/ mm^3^. Les adénopathies médiastinales, les atteintes moyennes, inferieures du champ pulmonaire et la miliaire étaient plus fréquentes chez les patients ayant un taux de lymphocytes T CD4< 200cell/ mm^3^. L’immunodépression sévère est significativement associée à la présentation radiographique atypique de la tuberculose.

## Abstract (en)

The aim was to describing the different radiographic features of pulmonary tuberculosis according to the degree of immunosuppression in HIV patients. We report a retrospective study of 80 untreated HIV-positive patients with pulmonary tuberculosis hospitalized in the Pneumo-Phthisiology Department in Brazzaville from January 2013 to January 2014. Our sample consisted of 44 women (55%) and 36 men (45%), the sex ratio was 0.81. The average age was 37.5 ± 9.17 years, the average CD4 count was 153.13 ± 86,6 cell/mm3. Sputum smear microscopy to detect acid-fast bacilli was positive in 75% of patients with CD4 cell count >200 cell/mm^3^. Mediastinal adenopathies, middle and lower lung field involvement and miliary were more frequent in patients with CD4 cell count < 200 cell/mm^3^. Severe immunosuppression is significantly associated with atypical radiographic presentation of tuberculosis.

## Introduction

La pandémie du Virus de l’Immunodéficience Humaine (VIH) et du Syndrome de l’Immunodéficience Acquise (SIDA) ont modifié les caractéristiques cliniques radiologiques de la tuberculose (TB) qui représente en Afrique la première affection opportuniste du VIH [1]. En effet, les deux infections interagissent l’une sur l’autre modifiant l’évolution de ces affections dont la mortalité est plus grande [1]. La prévalence de la coïnfection TB/VIH est en nette croissance dans le monde malgré la définition de nouvelles politiques de lutte contre ces deux affections intégrant les programmes de lutte contre la TB et le VIH. Elle est passée de 8,4% en 1994 à 13% en 2009 [2]. En république du Congo, la tuberculose reste un problème de santé publique majeure avec une prévalence d’environ 462 cas pour 100.000habitants, une incidence de 382 cas pour 100.000habitants par an. La coïnfection TB-VIH au Congo, est de 29% avec une prévalence VIH évaluée à 3,2% dans la population générale [3]. En Afrique sub-saharienne (ASS), notamment au Congo, la radiographie thoracique, compte tenu de sa disponibilité et de son faible coût, demeure l’examen de référence pour le bilan lésionnel de toute symptomatologie respiratoire [4]. Ainsi l’image de la TB thoracique chez les personnes vivant avec le VIH (PVVIH) est atypique et dépendrait du degré d’immunodépression. Contrairement aux sujets VIH négatifs, on note chez les sujets coinfectés TB/VIH, une augmentation des atteintes pleurales, ganglionnaires, la rareté des lésions cavitaires et une localisation apicale moins fréquente [5–8]. Au Centre Hospitalier Universitaire de Brazzaville, structure sanitaire de niveau 3 qui accueille la majorité des patients vivant avec le VIH, aucune étuden’a été réalisée portant sur l’impact de l’immunodépression sur la présentation radiographique de la tuberculose chez le sujet VIH positif. Il nous a paru opportun de mener cette étude qui avait pour objectif de décrire les aspects radiographiques de la tuberculose pulmonaire selon le degré de l’immunodépression chez les patients VIH positifs.

## Méthodes

### Type-cadre-période d’étude

Il s’est agi d’une étude transversale par analyse rétrospective de dossiers des patients hospitalisés au service de Pneumologie du CHU de Brazzaville(CHUB) du 1^er^ Janvier 2013 au 31décembre 2013, soit 12 mois.

### Patients

**Figure 1:**
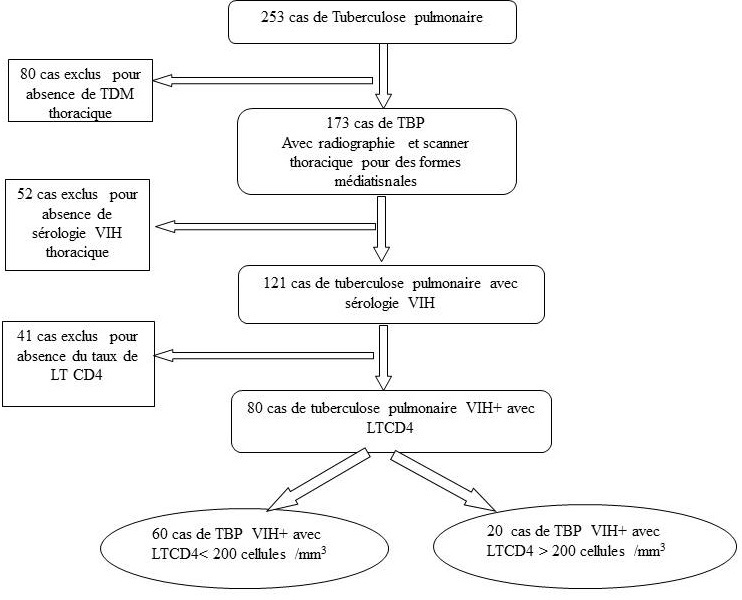
Inclusion des patients

Etaient inclus les patients hospitalisés au service de Pneumologie du CHUB pour tuberculose pulmonaire ayant une radiographie thoracique, un scanner thoracique en cas d’association d’atteintes médiatisnale et parenchymateuses, âgés d’au moins 16 ans, infectés par le VIH quelque soit le type, et dont le test de dépistage a été fait en per-hospitalisation; ne recevant pas le traitement antirétroviral. Nous n’avons pas inclus, des patients ayant une tuberculose pulmonaire sans sérologie VIH, patient ayant une atteinte médiatisnale associée sans scanner thoracique, (Figure 1). La taille de l’échantillona été l’effectif des patients répondant aux critères d’inclusion, hospitalisés durant la période de l’étude, soit un effectif de 80 patients. Puis les patients ont été scindés en deux groupes selon le taux de Lymphocyte T CD4(LTCD4). **Groupe 1: CD4≥ 200 cel/mm^3^; Groupe 2: CD4 = 200 cel/mm^3^**. Les variables étudiées étaientsociodémographiques (âge, sexe, profession, lieu de résidence, statut matrimonial) et para cliniques notamment la microscopie des expectorations à la recherche des bacilles acido-alcoolo-résistants, dosage du taux des LTCD4. Sur le plan radiographique, les lésions élémentaires recherchées étaient parenchymateuses, pleurales, et médiatisnales. Elles ont été analysées selon leur nombre, leur localisation, leur type et les associations lésionnelles ont été mentionnées.

### Définitions opérationnelles

Présentation radiographique typique: image nodulaire, ou infiltrative excavée à localisation apicale [9]; niveau socioéconomique bas: patient ayant un revenu inférieur au salaire interprofessionnel.

### Analyse des données

Les données ont été traitées au moyen du logiciel EPI info 3.3.2 (CDC Atlanta, USA) avec la détermination des statistiques descriptives et analytiques pour l’ensemble des sujets. Pour tous les tests, le seuil de signification a été fixé à 5%.

## Résultats

**Figure 2:**
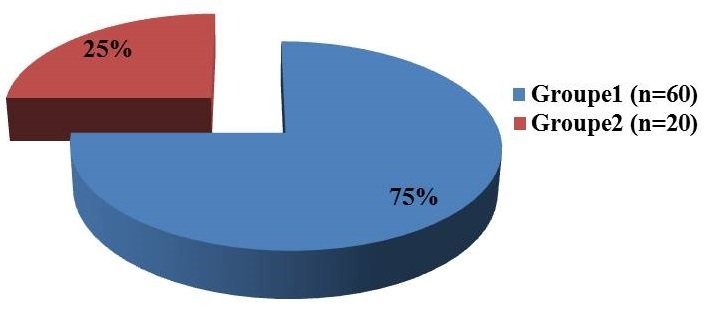
Répartition des patients selon le taux de Lymphocyte TCD4

Notre échantillonétait composé de 36 hommes et 44 femmes, soitun sex-ratio de 0,82. L’âge moyen d’âge denos patients était de 37.5± 9,17 ans avec des extrêmes allant de 21 ans à 72ans. Soixante dix (70) patients soit 87,5% de notre effectif avait un statut socioéconomique bas. La bacilloscopie des expectorations était positive chez 26cas/80 soit 32,9% et négatif 53cas/80 soit 67,1% (Figure 2). La moyenne du taux de LTCD4 était de 153,13± 86,6 cel/mm^3^ (1-355 cel/mm^3^). Selon le taux de LTCD4 60 patients soit 75% avaient un taux de LTCD4 < 200 cel/mm^3^ (Groupe1) et 20 patients avaient un taux de LTCD4 = 200 cel/mm^3^ (Groupe2).

### Données radiographiques

**Figure 3:**
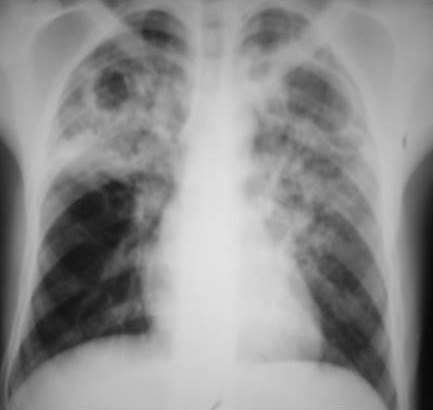
Infiltrats excavés des deux champs pulmonaires avec prédominance aux apex

Les 80 radiographies thoraciques de face, portaient toutes des anomalies. Ces anomalies concernaient dans 38,75% (31cas/80) le parenchyme, les lésions parenchymateuses étaient associées à une atteinte médiatisnale notamment des adénopathies (ADP) et pleurale dans les proportions respectives de 38,75%(31cas/80) et 22,5% (18cas/80) (Tableau 1). Les atteintes apicales étaient retrouvées dans 28,33% (17cas/60) dans le groupe 1 versus 60% (12/20) dans le groupe 2 (p=0,1). Les atteintes moyennes et inferieures étaient plus rencontrées dans le groupe 1 (39cas/80) avec un p<0,001 (Tableau 1). Les lésions bilatérales étaient retrouvées dans 73,33% (44cas/60) dans le groupe 1 versus35% (7cas/20) dans le groupe 2 (p<0,001). Les excavations étaient retrouvées dans 8,33%(5cas /60) dans le groupe1 et 85% (17cas/20) dans le groupe 2 avec un p<0,001 (Tableau 2). Les atteintes ADP médiatisnales représentaient 50% soit 30cas/60 dans le groupe 1 contre 5% (1cas/20) dans le groupe 2 (p<0,001). L’atteinte pleurale étaient retrouvées dans 26,67% soit 16cas/60 dans le groupe 1 versus 3,33% soit 2cas/20, p=0,004 (Tableau 2). La présentation radiographique était typique (Figure 3) dans 6,67% (4cas/60) dans le groupe 1 versus 90% (18 cas/20) dans le groupe 2 avec un p<0,001 (Tableau 2).

**Tableau 1:** Répartition de la localisation des images radiographiques selon le degré d’immunodépression

Localisation des lésions	Groupe 1		Groupe 2		p-value
	N	%	N	%	
**Atteinte apicale**	17	28,33	12	60	0,1
**Atteinte moyenne et inferieur**	**39**	**65**	**0**	**0**	**< 0,001**
**Bilatérale**	**44**	**73,33**	**7**	**35**	**< 0,001**
**Unilatérale**	18	30	13	65	0,11

Groupe 1: CD4< 200 cel/mm3; Groupe 2:CD4 ≥ 200 cel/mm3

**Tableau 2:** Répartition des types d’images radiologiques selon degré d’immunodépression

Type de lésion	Groupe 1		Groupe 2		p-value
	N	%	N	%	
**ADP médiastinales**	**30**	**50**	**1**	**5**	**0,001**
**Excavations**	**5**	**8,33**	**17**	**85**	**< 0,001**
**Atteintes pleurales associées**	**16**	**26,67**	**2**	**3,33**	**0,004**
**Miliaire**	7	11,67	0	0	0,12
**Présentation typique**	**4**	**6,67**	**18**	**90**	**< 0,001**

Groupe 1: CD4< 200 cel/mm3; Groupe 2:CD4 ≥ 200 cel/mm3**; ADP =** Adénopathie; **NB:** un patient pouvait avoir plus d’une lésion; Groupe 1(n=60), Groupe 2 (n=20)

## Discussion

Nous avons été confrontés à certaines difficultés inhérentes aux études rétrospectives. Il s’agit principalement de certaines informations manquantes dans les dossiers. Par ailleurs nous notons l’absence de scanner thoracique dans certains dossiers, de recherche de Bacilles Acido-Alcoolo-Résistant sur les autres prélèvements d’aspiration bronchique, du lavage broncho-alvéolaire, en dehors de l’expectoration spontanée. Nonobstantces écueils, notre étude a retrouvé une relation entre la présentation radiographique et le taux de LTCD4. Le taux LTCD4, est l’indicateur du statut immunitaire et du stade de l’infection à VIH [10–12]. La moyenne du taux de LTCD4 était de 153,13± 86,6 cel/mm. et la majorité de nos patients avait une immunodépression sévère (LTCD4<200cel/mm3). Cela est dû, au fait que le diagnostic de l’infection à VIH dans notre contexte se fait encore à des stades avancés de la maladie. A ce stade la tuberculose à une présentation clinique et para clinique atypique et le diagnostic de certitude nécessite des moyens plus invasifs qui le plus souvent font défaut dans note pratique. Un accent particulier devrait être mis sur le test dépistage volontaire de l’infection à VIH. L’immunodépression sévère était significativement associée aux ADP médiastinales. Les atteintes médiatisnales (ADP) étaient plus retrouvéeschez les patients ayant une immunodépressionsévère. Cette fréquence s’expliquerait par l’envahissement secondaire du relais ganglionnaire thoracique [13], mais aussi par l’association possible avec un lymphome non hodgkinien fréquemment rencontré [14]. D’autres lésions atypiques telle que les atteintes du parenchyme pulmonaire moyen, inferieur et la miliaire étaient retrouvés chez les patients ayant un taux de LTCD4 < 200cel/mm^3^. Ce constat est retrouvé dans plusieurs études [15–17]. L’atteinte apicale commune aux patients négatifs au VIH et positifs ayant un fort taux de LTCD4, était plus retrouvée chez les patients ayant un taux de LTCD4 > 200cel/mm^3^ mais cela n’était pas significatif. Les lésions parenchymateuses chez les sujets ayant une immunodépression sévère sont en règle extensives, représentées par des infiltrats diffus, non excavées. En effet 73,33% des patients ayant un taux de LTCD4 < 200cel/mm^3^ avaient des lésions bilatérales et diffuses.

**Figure 4:**
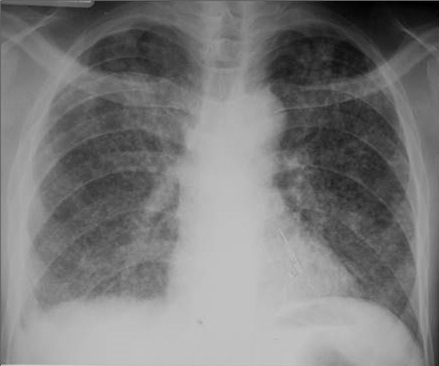
Opacités micronodulaires disséminées dans les deux champs pulmonaires (Miliaire)

La miliaire (Figure 4), une des formes graves de la tuberculose étaient retrouvées uniquement chez les sujets ayant une immunodépression sévère. Cette distribution s’expliquerait par le niveau d’immunodépression qui favoriserait la diffusion des lésions. Notre étude rapporte une fréquence élevée des excavations chez les patients ayant un taux de LTCD4 > 200cel/mm^3^ (p<0,001). En effet l’immunité cellulaire efficace, engendre une réaction antigène-anticorps lors de l’agression par le *Mycobaterium Tuberculosis* à l’origine de la constitution d’un granulome inflammatoire. La nécrose tissulaire secondaire due au *Mycobaterium Tuberculosis*avec excavation ultérieure est à l’origine des cavernes tuberculeuses. Ces mécanismes de défense contre l’infection tuberculeuse sont perturbés avec altération de l’immunité à médiation cellulaire [18–20].

## Conclusion

Notre étude a montré une relation entre les images radiographiques de la tuberculose et le degré de l’immunodépression chez le sujet VIH positif. Ainsi une immunodépression profonde engendre des lésions radiographiques plus étendues et peu typiques des aspects anciennement décrits dans la tuberculose.

### Etat des connaissances actuelle sur le sujet

- La prévalence de la coïnfection TB/VIH est en nette croissance dans le monde;
- Les deux infections interagissent l’une sur l’autre modifiant l’évolution de ces affections dont la mortalité est plus élevée;
- La pandémie du Virus de l’Immunodéficience Humaine (VIH) et du Syndrome de l’Immunodéficience Acquise (SIDA) a modifié les caractéristiques cliniques radiologiques de la tuberculose (TB).

### Contribution de notre étude à la connaissance

- Notre étude apporte les données scientifiques car aucune étude n’a été réalisée portant sur l’impact de l’immunodépression sur la présentation radiographique de la tuberculose chez le sujet VIH positif au Congo;
- Une relation entre la présentation radiographique et le taux de LTCD4;
- L’immunodépression sévère était significativement associée aux ADP médiastinales.
